# Modic changes in the lumbar spine and their association with body composition, fat distribution and intervertebral disc height – a 3.0 T-MRI study

**DOI:** 10.1186/s12891-016-0934-x

**Published:** 2016-02-19

**Authors:** Andrew J. Teichtahl, Donna M. Urquhart, Yuanyuan Wang, Anita E. Wluka, Richard O’Sullivan, Graeme Jones, Flavia M. Cicuttini

**Affiliations:** Department of Epidemiology and Preventive Medicine, School of Public Health and Preventive Medicine, Monash University, Alfred Hospital, Melbourne, VIC 3004 Australia; Baker IDI Heart and Diabetes Institute, Commercial Road, Melbourne, VIC 3004 Australia; Healthcare Imaging Services, Epworth Hospital, Richmond, Melbourne, VIC 3121 Australia; Department of Medicine, Central Clinical School, Monash University, Melbourne, VIC 3004 Australia; Menzies Research Institute, Private bag 23, Hobart, TAS 7000 Australia

**Keywords:** Lumbar spine, Intervertebral disc, Modic, Fat mass, Fat free mass, Android, Gynoid, Height, Body composition, MRI, Adiposity

## Abstract

**Background:**

Vertebral endplate (Modic) abnormalities are important structural lesions in the spine, but their association with body composition and fat distribution have not been examined. Moreover, no study has examined whether Modic change are related to other structural features of low back pain, such as reduced intervertebral disc height.

**Methods:**

Seventy-two community-based individuals not selected for low back pain had lumbar vertebral Modic change and intervertebral disc height assessed from MRI. Dual energy x-ray absorptiometry measured body composition and fat distribution.

**Results:**

The predominance of Modic change was type 2. Modic change was associated with an increased fat mass index (OR 1.20, 95 % CI 1.01 to 1.43), and tended to be associated with a reduced fat-free mass index (OR 0.62, 95 % CI 0.37 to 1.03, *p* = 0.07). While an increased percentage of gynoid fat was associated with a reduced risk (OR 0.62, 95 % CI 0.43 to 0.89), an increased percentage of android fat was associated with an increased risk of Modic change (OR 2.11, 95 % CI 1.18 to 3.76). Modic change was also associated with reduced intervertebral disc height at L2/3, L4/5 and L5/S1 (OR range 1.4 to 1.8; all *p* ≤ 0.03).

**Conclusion:**

Modic type 2 change is associated with reduced intervertebral disc height and an increased fat mass index. Whereas gynoid fat distribution protected against Modic type 2 change, an android pattern increased the risk of this lesion. Modic type 2 change, which histologically represent fat replacement, might have a metabolic component to its aetiology.

## Background

The advent of magnetic resonance imaging (MRI) has enabled comprehensive structural assessment of the spine, providing new insights into the structural features associated with spinal pathology and non-specific low back pain. Recently, there has been particular interest in lumbar vertebral body marrow and endplate lesions, termed “Modic change” [1]. Three Modic changes have been classified, each with different histopathologic correlates. Modic type 1 change represents bone marrow oedema and inflammation; Modic type 2 change represents marrow ischemia and the conversion of normal red haemopoietic bone marrow into yellow fatty marrow [1]; and Modic type 3 change is rare and represents of subchondral bone sclerosis [2].

Although the histopathologic correlates of Modic change are becoming better elucidated, the clinical significance of Modic change remains the subject of investigation. Modic type 1 lesions in the lumbar spine are strongly associated with low back pain [3–7] and instability [3, 8]. With the passage of time, a large proportion of Modic type 1 change converts to type 2 [1, 4, 7, 9]. Modic type 2 change is less strongly associated with low back pain [3–6]. Nevertheless, Modic type 2 change is more common than type 1 [1, 2, 5, 10–12], and more frequent among individuals with degenerative disc disease [3, 13, 14]. Since Modic type 2 change is characterized histologically by fatty infiltration [1], it is possible that this lesion is related to adiposity. Although not widely examined, BMI has not been shown to be associated with Modic change [12]. Nevertheless, BMI cannot discriminate adipose from non-adipose tissue. No study has examined whether body composition and fat distribution are associated with Modic change.

Although past studies have demonstrated that Modic type 2 change occurs at sites of degenerative disc disease [1, 2, 10, 11], the definition of degenerative disc disease is broad and incorporates a multitude of radiological features including disc protrusion, herniation and reduced intervertebral disc height. No study has examined the associations between Modic change and individual radiological features of disc degeneration, such as intervertebral disc height.

The aim of this cross-sectional study was to determine whether body composition is a risk factor for Modic change in community-based adults and whether Modic change is associated with intervertebral disc height. We hypothesized that i) adiposity is a risk factor for Modic change; and ii) Modic change is associated with reduced intervertebral disc height.

## Methods

### Participants

Seventy-two community-based individuals recruited through local media and weight loss clinics were examined as part of a study of obesity and musculoskeletal health. Participants were not required to have low back pain or a history of low back for inclusion in the study. Exclusion criteria included malignancy, significance systemic condition, or inability to understand English. Participants gave written informed consent. The study was approved by the Human Research and Ethics Committees of the Alfred Hospital, Monash University, Austin Health and the University of Melbourne.

### Magnetic resonance imaging

MRI was performed using a 3.0-T magnetic resonance unit (MAGNETOM Verio, A Tim System; Siemens, Erlangen, Germany) in 2012. The participant was positioned in supine and the following scans were performed: [1] sagittal T1 images from T12 to the sacrum (time to recovery 670 ms; time to echo: 12 ms, slice thickness: 4 mm), [2] sagittal T2 images from T12 to sacrum (time to recovery: 3000–3600 ms; time to echo: 87–114 ms, slice thickness: 4 mm), and [3] axial T2 images from L1 to L3 and L3 to S1 (time to recovery: 3000–3600 ms; time to echo: 87–114 ms, slice thickness: 4 mm).

Modic change were assessed using the original classification by Modic et al. [1, 2] which consists of 3 type:

- Type 1: hypointense on T1 and hyperintense on T2 images
- Type 2: hyperintense on T1 and isointense/hyperintense on T2 images
- Type 3: hypointense on both T1 and T2 images

**Fig. 1:**
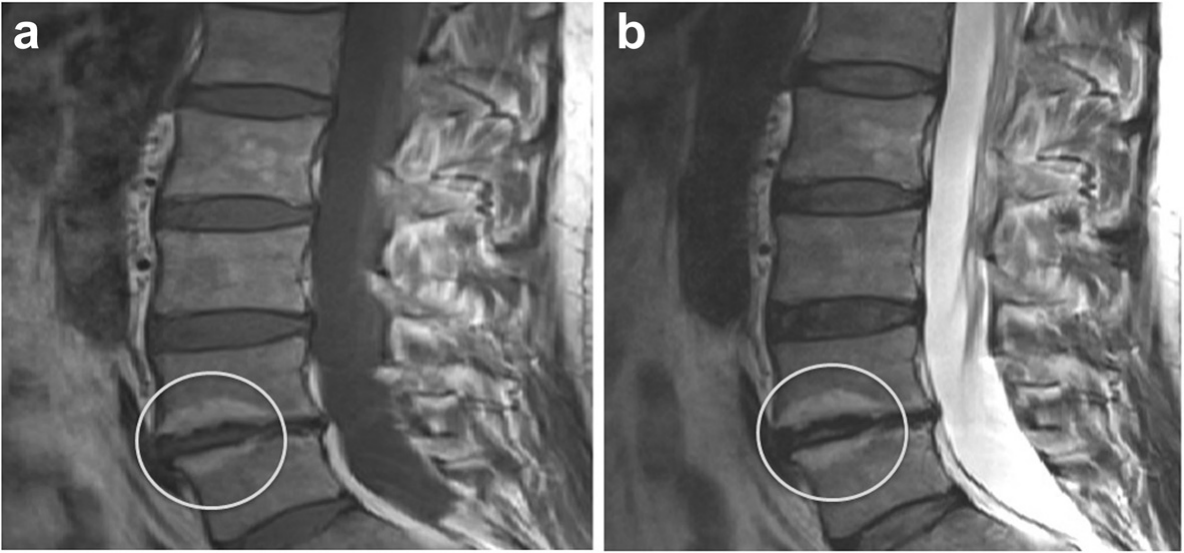
An example of Modic type 2 change in the vertebrae adjacent to the L4/5 intervertebral disc, which also demonstrates reduced disc height. **a** – T1-weighted image, **b** – T2-weighted image

Images were assessed in the sagittal plane. Sixty randomly selected images were reassessed by an independent observer (AT) for Modic change. The inter-observer reliability (ICC) was found to be 0.74. An example of Modic type 2 change is shown in Fig. 1.

Intervertebral disc height of the lumbar spine was measured on mid-sagittal MR images from the middle of the superior border of the disc to the middle of the inferior border of the disc with the inclusion of both end-plates. One trained observer was taught to measure disc height by a radiologist experienced in musculoskeletal MRI, measured the disc height in duplicate, 1 week apart, blinded to the characteristics of the participants. The intra-rater reliability of the disc height measures at each vertebral level was high, with intra-class correlation coefficients (ICCs) ranging from 0.94 to 0.98.

### Anthropometric data

Height was measured to the nearest 0.1 cm using a stadiometer. Weight was measured to the nearest 0.1 kg using a single pair of electronic scales. BMI (kg m^−2^) was calculated.

### Body composition and fat distribution

Body composition was measured using dual energy x-ray absorptiometry (GE Lunar Prodigy, using operating system version 9; GE Healthcare, United Kingdom) between 2006 and 2008. The machine has a weight limit of approximately 130 kg. Fat mass index was calculated by fat mass (kg)/height (m^2^) while fat free mass index was calculated by fat free mass (kg)/height (m^2^). Standard regional analyses were used to measure total body, android, and gynoid fat mass, as well as total body lean tissue mass. Android fat mass relates to the distribution of excess fat predominantly around the abdomen. Gynoid fat mass relates to the distribution of excess fat predominantly around the hips, thighs, and buttocks, respectively. The percentage of android and gynoid fat were calculated by dividing each respective measure in kg, but the total fat mass in kg.

### Low back pain and disability

The Chronic Pain Grade Questionnaire was administered to obtain information on low back pain intensity and disability over the past 6 months at the time of MRI. The Chronic Pain Grade Questionnaire is a reliable and valid instrument for use in population surveys of low back pain [15, 16]. The questionnaire includes seven questions from which a pain intensity score (0–100) was calculated. To gauge the degree of pain intensity in the community-based cohort, participants were classified into three groups based on their pain intensity score; no pain (=0), low pain intensity (<50) and high pain intensity (≥50) as previously described [15, 16].

### Statistical analyses

Binary logistic regression was used to examine the relationships between lumbar intervertebral disc height and Modic change at each individual level of the lumbar spine, as well as the association between Modic change at any level of the lumbar spine and the total and average intervertebral disc height of the lumbar spine. Adjustment for age, gender, height and weight were made. Binary logistic regression was used to examine the relationships between Modic change at any level of the lumbar spine and body composition and fat distribution. The analyses of fat mass index and fat distribution measures were adjusted for age, gender, and fat free mass index. The analysis of fat free mass index was adjusted for age, gender, and fat mass index. An additional regression equation was created whereby all analyses were further adjusted for the average intervertebral disc height of the lumbar spine. A p-value of less than 0.05 (two-tailed) was regarded as statistically significant. All analyses were performed using the SPSS statistical package (standard version 20.0 SPSS, Chicago, IL, USA).

## Results

Seventy-two participants had MRI data available, of which 57 had body composition and fat distribution data. There were no significant differences between the 57 participants and the remaining 15 participants who did not have body composition and fat distribution data in terms of age (49.7 years versus 48.2 years, *p* = 0.36), gender (73.7 % versus 53.3 % female; *p* = 0.17), and intervertebral disc height at any level of the lumbar spine (*p* ≥ 0.47). People who did not have body composition and fat distribution data were more obese than those that did (BMI 33.5 kgm^−2^ versus 28.6 kgm^−2^, *p* = 0.04). Characteristics of the 57 participants are shown in Table 1. The majority of subjects in the study were female (73.7 %), while the average BMI was in the overweight category (28.6 kgm^−2^). The predominance of participants had no or low levels of chronic back pain (75.4 %) according to the chronic pain grade questionnaire. The predominance of Modic change was type 2, with only 1 participant demonstrating Modic type 1 change. Eighteen subjects demonstrated a Modic change type 2 in the lumbar spine, with the majority of these located at L4 (19.3 % of cohort) and L5 (21.1 % of cohort).

**Table 1:** Subject demographics (*n* = 57)

Age (years)	49.7 (7.9)
Gender (n, % female)	42 (73.7)
Weight (kg)	78.3 (18.4)
Height (cm)	166 (9.11)
BMI (kgm^−2^)	28.6 (6.7)
*Chronic low back pain grade*	
*0 - None*	13 (22.8)
*1- Low*	30 (52.6)
*2 - High*	14 (24.6)
Disc height (mm)	
*L1/2*	9.7 (1.7)
*L2/3*	11.1 (2.0)
*L3/4*	11.8 (2.2)
*L4/5*	11.5 (2.5)
*L5/S1*	10.4 (2.9)
Average disc height	10.9 (1.6)
Total disc height	54.8 (8.2)
Modic grade 2 (%)	
*L1*	1 (1.8)
*L2*	0 (0)
*L3*	3 (5.3)
*L4*	11 (19.3)
*L5*	12 (21.1)
*S1*	5 (8.8)
Modic type 2 change in vertebrae adjacent to disc	
*L1 or L2*	1 (1.8)
*L2 or L3*	4 (7.0)
*L3 or L4*	12 (21.2)
*L4 or L5*	16 (28.1)
*L5 or S1*	12 (21.1)
Any Modic change in lumbar vertebrae	18 (31.6)
DEXA measures	
*Fat mass index (kg m* ^*−2*^ *)*	10.8 (6.1)
*Fat free mass index (kg m* ^*−2*^ *)*	18.1 (2.6)
*% Gynoid fat*	20.2 (3.7)
*% Android fat*	8.8 (1.9)

Results presented as mean (standard deviation) unless otherwise stated

The associations between lumbar intervertebral disc height and Modic change in the adjacent vertebrae are shown in Table 2. After adjusting for age, gender, weight, and height, a reduction in disc height at either L2/3, L4/5 and L5/S1 were associated with the presence of Modic change in the adjacent vertebrae (OR range 1.4 to 1.8; 95 % CI range from 1.1 to 2.9; all *p* ≤ 0.03). For every 1 mm reduction in the average intervertebral disc height for the lumbar spine, there was a 1.6 times increased risk for the presence of any Modic change (95 % CI 1.1 to 2.3, *p* = 0.01). Similarly, for every 1 mm reduction in the total intervertebral disc height for the lumbar spine, there was a 1.1 times increased risk for the presence of Modic change (95 % CI 1.0 to 1.2, *p* = 0.01).

**Table 2:** The associated risk of Modic change in the lumbar spine as intervertebral disc height (mm) reduces

Disc level	Risk of Modic change in the vertebrae adjacent to that disc			
	Univariate OR 95 % CI	*P*	Multivariate OR 95 % CI	*P*
L1/2	1.5 (0.9, 2.3)	0.11	1.5 (0.8, 3.0)	0.22
L2/3	1.7 (1.1, 2.7)	0.01	1.8 (1.1, 2.9)	0.03
L3/4	1.1 (0.9, 1.4)	0.29	1.1 (0.8, 1.4)	0.64
L4/5	1.3 (1.1, 1.7)	0.007	1.4 (1.1, 1.8)	0.008
L5/S1	1.4 (1.1, 1.7)	0.001	1.4 (1.1, 1.8)	0.001
	Risk of Modic change in the lumbar spine			
	Univariate OR 95 % CI	*P*	Multivariate OR 95 % CI	*P*
Average disc height (L1-S1)	1.5 (1.1, 2.0)	0.02	1.6 (1.1, 2.3)	0.01
Total disc height (L1-S1)	1.1 (1.0, 1.2)	0.02	1.1 (1.0, 1.2)	0.01

Adjusted for age, gender, height and weight

The associations between Modic change and body composition and fat distribution are shown in Table 3. After adjusting for age, gender and fat free mass index, an increased fat mass index was associated with an increased risk for Modic change in the lumbar spine (OR 1.20, 95 % CI 1.01 to 1.43, *p* = 0.03). This relationship tended toward significance after adjusting for the average intervertebral disc height in the lumbar spine (*p* = 0.05). In contrast, increased fat free mass index tended toward being associated with a reduced risk for Modic change after adjusting for age, gender, fat mass index and the average lumbar spine intervertebral disc height (OR 0.62, 95 % CI 0.36 to 1.05, *p* = 0.08). Whereas an increased percentage of gynoid fat was associated with a reduced risk of lumbar spine Modic change (OR 0.62, 95 % CI 0.43 to 0.89, *p* = 0.01), an increased percentage of android fat was associated with an increased risk of lumbar spine Modic change (OR 2.11, 95 % CI 1.18 to 3.76, *p* = 0.01).

**Table 3:** The associations between body composition and fat distribution and the risk for Modic changes in the lumbar spine

	Univariate OR (95 % CI)	*P*	Multivariate^a^ OR (95 % CI)	*P*	Multivariate^b^ OR (95 % CI)	P
Fat measures						
Fat mass index	1.08 (0.97, 1.19)	0.17	1.20 (1.01, 1.43)	0.03	1.19 (1.00, 1.42)	0.05
% gynoid fat	0.81 (0.66, 1.00)	0.05	0.62 (0.44, 0.88)	0.007	0.62 (0.43, 0.89)	0.01
% android fat	1.43 (1.00, 2.05)	0.05	2.14 (1.23, 3.73)	0.008	2.11 (1.18, 3.76)	0.01
Lean measures						
Fat free mass index	0.90 (0.67, 1.21)	0.49	0.62 (0.37, 1.03)	0.07	0.62 (0.36, 1.05)	0.08

^a^Fat free mass index - adjusted for age, gender and fat mass index; All fat measures - adjusted for age, gender and fat free mass index

^b^Average disc height from L1-S1 added to regression equation

## Discussion

This study of community-based adults demonstrated that Modic type 2 change is by far the most prevalent Modic change, and is associated with reduced intervertebral disc height of the lumbar spine. While increased fat mass was associated with a higher risk of Modic type 2 change, increased fat free mass tended to protect against this lesion. Moreover, distribution of body fat was important; with a gynoid pattern protecting against Modic type 2 change, and an android pattern of fat distribution increasing the risk of this lesion. Such findings suggest that Modic type 2 change, which histologically represents fat replacement of marrow, might have a metabolic component in its aetiology.

This study supports previous reports of a higher prevalence of Modic type 2 change at the L4-5 and L5-S1 level in the general community [5, 12, 17]. Moreover, past studies have demonstrated that Modic change occurs at sites of degenerative disc disease [1, 2, 10, 11]. Nevertheless, the definition of degenerative disc disease is broad and incorporates a multitude of radiological features including disc protrusion, herniation and reduced intervertebral disc height. No study has examined the associations between Modic change and individual radiological features of disc degeneration. We have used intervertebral disc height as a measure of disc degeneration. We recently demonstrated a negative dose–response relationship between increasing severity of disc degeneration with a reduction in intervertebral disc height [18]. The utility of intervertebral disc height as a quantitative and continuous measure enabled us to sensitively examine the relationships between intervertebral disc height and Modic lesions. For instance, we have shown that a reduction in intervertebral disc height is associated with Modic type 2 change in the adjacent vertebrae of the low lumbar spine, as well as the total and average intervertebral disc height of the lumbar spine. For every 1 mm reduction in the average disc height of the lumbar spine, there was a 1.6 times (95 % CI 1.1 to 2.3) increased risk of Modic change in the lumbar spine. Since the intervertebral disc abutting the vertebral endplates is comprised of avascular fibrocartilage, it is possible that the histological features of fatty replacement of the bone marrow that characterizes progressive Modic change [1, 2] impedes nutritional support to the intervertebral disc with resultant disc degeneration and loss of disc height. Alternatively, reduced intervertebral disc height may attenuate the cushioning effect and load redistribution to adjacent vertebrae, resulting in Modic change. The cross-sectional design of this study cannot however determine whether reduced intervertebral disc height is a cause or result of Modic change and longitudinal studies are required to explore a causal relationship.

Although not widely examined, BMI has not been shown to be associated with Modic change [12]. Nevertheless, BMI cannot discriminate adipose from non-adipose tissue. No previous study has examined whether body composition and fat distribution are associated with spinal structural change. In the current study, we observed an increased risk of Modic type 2 change in relation to greater fat mass but a reduced risk for this lesion associated with greater fat free mass. Such findings demonstrate the potential importance of body composition in the pathogenesis of structural abnormalities in the low back. Additionally, this study has also shown that the distribution of body fat is a determinant of Modic type 2 change. Whereas a gynoid pattern of fat distribution protected against the presence of Modic change, an android pattern increased the risk of Modic change in the lumbar spine. Android fat accumulates in the upper and central body, particularly the abdomen, while gynoid fat accumulates in the lower body, particularly the hips and thighs.

Compared with the gynoid pattern, the android pattern of fat distribution is considered to be more metabolically active and has been associated with increased risk of cardiovascular disease [19, 20], abnormal lipid profiles and diastolic blood pressure [21]. It is also becoming increasingly recognized that the android pattern of fat distribution is an important determinant of the burden of musculoskeletal diseases and a recent study has shown that android fat is strongly associated with foot pain and disability [22]. Moreover, android fat has been associated with structural abnormalities at the knee, such as cartilage defects [23] and reduced cartilage volume [24]. Whereas non-specific back pathology has typically been considered to have a strong biomechanical component to its aetiology, these data provide evidence that Modic change may be related to metabolic processes. Nevertheless, we cannot exclude a biomechanical contribution to Modic change. It is possible that excess mass in the trunk increases axial loads, leading to structural abnormalities such as Modic change. Nevertheless, this biomechanical mechanism would not explain the protective association between gynoid fat distribution and Modic change.

The mechanism accounting for the relationship of body composition and fat distribution with Modic change is however, speculative. Histologically, Modic type 2 change is representative of fatty replacement of the bone marrow [1], and may therefore be closely linked to adiposity. Although previously considered a passive store of energy, adipose tissue is now recognized as a highly active metabolic structure that contributes to systemic inflammation, secreting a host of pro-inflammatory cytokines including tumor necrosis factor (TNF) and interleukin-6 (IL-6) [25]. Both TNF-alpha and IL-6 have been shown to be predominantly derived from the adipose bed located within the trunk and abdomen [26]. TNF immunoreactive cells have been shown to be present in greater number in people with Modic change, and particularly Modic type 1 change [27]. Whereas Modic type 1 change is considered inflammatory in nature and a source of low back pain, the natural history of Modic change in the lumbar spine suggests that the majority of type 1 lesions progress to type 2 [3–7]. Since body composition and fat distribution data was collected approximately 4 to 6 years prior to MRI assessment, it is possible that the systemic inflammation imparted by adiposity resulted in type 1 Modic change that, with the passage of time, progressed to Modic type 2 change seen in this study. Longitudinal studies may help to better elicit the natural history between body composition, fat distribution and the natural history of Modic change.

Our cross-sectional study had a modest sample size and mainly comprised females (73.7 %). A larger study with a more even gender distribution is required to substantiate the generalizability of our findings. Moreover, longitudinal studies will help to determine any causal relationships between body composition, fat distribution and Modic change, as well as the interaction between Modic change and intervertebral disc degeneration. Fifty-seven of 72 participants had body composition and fat distribution measures performed. The baseline characteristics of the 15 subjects without these data were not different from the 57 with these data available, other than people who did not have absorptiometry (*n* = 15) being significantly more obese (BMI 33.5 kgm^−2^ versus 28.6 kgm^−2^, *p* = 0.04). The loss of more obese people not having absorptiometry would likely have reduced the chance of showing significant relationships between measures of body composition and fat distribution and MRI abnormalities. Additionally, measures of body composition and fat distribution were taken 4 to 6 years prior to MRI assessment of the lumbar spine. Since we have shown that the fat mass index was associated with an increased risk of Modic type 2 lesions, any weight loss that may have occurred from recruiting in part from weight loss clinics, would have underestimated associations. This is a strength of the study since it implies that an android pattern of fat distribution precedes Modic change. Reverse causality whereby Modic change predated an android pattern of fat distribution is less likely in a community-based population not recruited on the basis of established back pain and disability that could have imparted activity restriction and a subsequent change in body composition. Indeed, a large population study of people aged 15 to 98 years demonstrated that the natural history of fat mass is to increase with ageing, while fat free mass tends to reduce once reaching its peak between the ages of 35 to 54 years [28]. Such findings may account for the growing prevalence of low back pain seen with ageing [29]. Moreover, Modic change in this study was predominantly type 2, with only one participant having a type 1 change. Whether body composition and fat distribution are associated with Modic type 1 lesions requires investigation and may be best explored by selectively recruiting people with low back pain at the time of MRI. Finally, although the disc height at the L3/4 level was not significantly associated with Modic, this may be a reflection of the low prevalence of Modic lesions at this level. Indeed, Modic lesions were more common at L4 (19.3 %) and L5 (21.1 %), and less common at L3 (5.3 %). The lower prevalence at L3 has likely reduced the ability of this study to demonstrate associations at this level. Future studies would benefit from a larger sample size to address such issues.

## Conclusions

This study has demonstrated Modic type 2 change, which is associated with reduced intervertebral disc height of the lumbar spine, is positively associated with fat mass while fat free mass tended to protect against Modic change. Moreover, distribution of body fat was important and whereas a gynoid pattern protected against Modic change, an android pattern of fat distribution increased the risk for Modic change. Such findings suggest that Modic type 2 change, which histologically represents fatty replacement of bone marrow, may have a metabolic component in its aetiology.

## Abbreviations

BMI: Body mass index
CI: Confidence interval
ICC: Intra-class correlation coefficient
IL: Interleukin
MRI: Magnetic resonance imaging
OR: Odds ratio
TNF: Tumor necrosis factor
